# Supplementary material for: Genetic and Pathogenic Analysis of a Novel Porcine Epidemic Diarrhea Virus Strain Isolated in the Republic of Korea
Source: Viruses. 2024 Jul 10;16(7):1108. doi: 10.3390/v16071108 (PMC11281356; doi:10.3390/v16071108)
Supplement: Supplementary file 1 [file viruses-16-01108-s001.zip › 3. 24.05.20 viruses supplementary data.pdf]

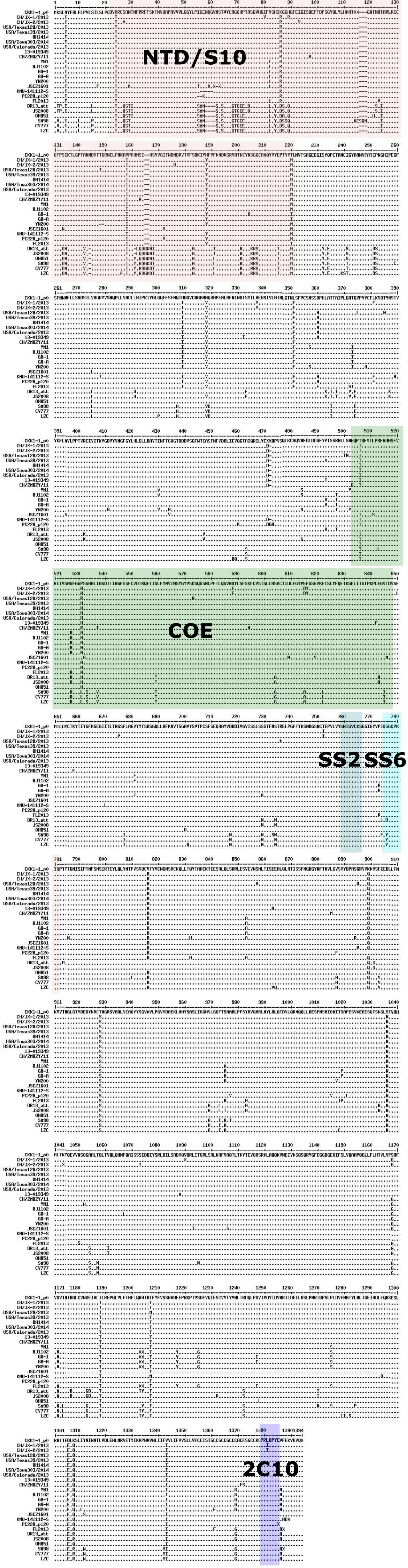
NTD/S10
COE
SS2
SS6
2C10
