# Supplementary material for: Genetic and Pathogenic Analysis of a Novel Porcine Epidemic Diarrhea Virus Strain Isolated in the Republic of Korea
Source: Viruses. 2024 Jul 10;16(7):1108. doi: 10.3390/v16071108 (PMC11281356; doi:10.3390/v16071108)
Supplement: Supplementary file 1 [file viruses-16-01108-s001.zip › 2. 2024.05.20 Viruses supplementary data.pdf]

Supplementary Materials

Figure S1. Comparison of deduced spike protein between CKK1-1 and reference strains. Alignment of deduced spike protein. The N-terminal domain (NTD) and major neutralizing epitopes (COE, SS2, SS6, 2C10) was represented by color box. The deletion of amino acid was indicated by "-".
